# Gallic Acid Enriched Fraction of *Phyllanthus emblica* Potentiates Indomethacin-Induced Gastric Ulcer Healing via e-NOS-Dependent Pathway

**DOI:** 10.1155/2012/487380

**Published:** 2012-08-26

**Authors:** Ananya Chatterjee, Sirshendu Chatterjee, Angshuman Biswas, Sayanti Bhattacharya, Subrata Chattopadhyay, Sandip K. Bandyopadhyay

**Affiliations:** ^1^Department of Biochemistry, University College of Medicine, I.P.G.M.E&R, 244B A.J.C. Bose Road, West Bengal, Kolkata 700020, India; ^2^Central Research Laboratory, Department of Biochemistry, KPC Medical College and Hospital, 1F Raja S.C. Mullick Road, Jadavpur, West Bengal, Kolkata 700032, India; ^3^Department of Pharmaceutical Chemistry, Central Drugs Laboratory, 3 Kyd Street, Kolkata-700016, India; ^4^Bio-Organic Division, Bhabha Atomic Research Centre, Mumbai 400085, India

## Abstract

The healing activity of gallic acid enriched ethanolic extract (GAE) of *Phyllanthus emblica* fruits (amla) against the indomethacin-induced gastric ulceration in mice was investigated. The activity was correlated with the ability of GAE to alter the cyclooxygenase- (COX-) dependent healing pathways. Histology of the stomach tissues revealed maximum ulceration on the 3rd day after indomethacin (18 mg/kg, single dose) administration that was associated with significant increase in inflammatory factors, namely, mucosal myeloperoxidase (MPO) activity and inducible nitric oxide synthase (i-NOS) expression. Proangiogenic parameters such as the levels of prostaglandin (PG) E_2_, vascular endothelial growth factor (VEGF), hepatocyte growth factor (HGF), von Willebrand Factor VIII, and endothelial NOS (e-NOS) were downregulated by indomethacin. Treatment with GAE (5 mg/kg/day) and omeprazole (3 mg/kg/day) for 3 days led to effective healing of the acute ulceration, while GAE could reverse the indomethacin-induced proinflammatory changes of the designated biochemical parameters. The ulcer healing activity of GAE was, however, compromised by coadministration of the nonspecific NOS inhibitor, *N*-nitro-L-arginine methyl ester (L-NAME), but not the i-NOS-specific inhibitor, L-*N6*-(1-iminoethyl) lysine hydrochloride (L-NIL). Taken together, these results suggested that the GAE treatment accelerates ulcer healing by inducing PGE_2_ synthesis and augmenting e-NOS/i-NOS ratio.

## 1. Introduction

In Indian Ayurvedic system of medicine, *Phyllanthus emblica* (syn: *Emblica officinalis, *family: Euphorbiaceae), is valued for its remarkable therapeutic activity against different diseases. According to belief in ancient Indian mythology, *P. emblica* (family: Euphorbiaceae) is the first tree to be created in the universe [1]. Its fruits, commonly known as “amla” are rich sources of vitamin C, various hydrolysable tannins such as emblicanin A and B, punigluconin, pedunculagin, galloellagitannins, and flavones like rutin [2]. However, gallic acid is the major key bioactive component having excellent antioxidative [3], antimutagenic [4], anticancer, and antiviral activities [5, 6]. 

The nonsteroidal anti-inflammatory drugs (NSAIDs) are one of the most widely prescribed drugs in the world and are extensively used to alleviate clinical cases of pain and inflammation [7], prevention and treatment of ischemic heart disease [8], and neoplasia [9]. However, these drugs are well known for stomach ulceration and delayed ulcer healing properties [10]. Currently, the use of NSAIDs accounts for approximately 25% of gastric ulcer cases with an upward trend [11, 12]. Apart from the systemic activity which mainly involves inhibition of cyclooxygenases (COXs), reduced prostaglandin synthesis, and impaired prostaglandin-(PG-) mediated angiogenesis, the NSAIDs also affect the COX-independent mechanisms especially the nitrogen-metabolizing enzymes that are also key contributors in wound healing [13, 14]. Despite recent advances, adequate remedy for the NSAID-induced gastropathy remains elusive. The World Health Organization (WHO) has stressed the need to develop drugs from plant origin, which will be inexpensive, accessible particularly to the rural people in the developing countries, and show less/no side effects. Recently, we have shown that the ethanolic extract of amla has significant healing activity against indomethacin-induced gastric ulceration in mice [15] by its antioxidant action. In a separate study, we have also established gallic acid as the active principle of the amla extract and explained the healing action in terms of its immunomodulatory action [16]. Hence, in the present study we fractionated the ethanolic extract of amla to prepare the gallic acid-enriched extract (designated as GAE throughout the paper) and tested its gastric ulcer healing activity in mice. Because PG [17], endothelial NOS (e-NOS), and nitric oxide (NO) (but not inducible nitric oxide synthase (i-NOS)), derived (NO)) are crucial for gastric ulcer healing, we compared the status of the mucosal i-NOS/e-NOS ratio as well as the NO and PGE_2_ levels in the ulcerated and GAE-treated mice. In addition, the myeloperoxidase(MPO) activity, expression of growth factors (vascular endothelial growth factor (VEGF) and hepatocyte growth factor (HGF)) in the gastrointestinal tract, and angiogenesis (in terms of von Willebrand Factor VIII) [14, 18] that facilitate tissue formation and tissue remodeling were investigated in the ulcerated and treatment groups. Finally the healing property of GAE was correlated with its ability to modulate the above parameters. However, we also assessed the ulcer healing in the pure gallic acid-treated mice by tissue histology to reaffirm that it is the active principle of the amla extract. 

## 2. Material and Methods

### 2.1. Chemicals and Reagents

Indomethacin, omeprazole, 5-bromo-4-chloro-3-indolyl phosphate (BCIP), nitroblue tetrazolium (NBT), Tween-20, Bradford reagent, hexadecyltrimethylammonium bromide (HTAB), L-*N*6-(1-iminoethyl) lysine hydrochloride (L-NIL), *N*-nitro-L-arginine methyl ester (L-NAME), and gallic acid were purchased from Sigma Chemical Co, St. Louis, MO, USA. Other chemicals used were ethanol and methanol (E. Merck, Mumbai, India); 35% hydrogen peroxide (H_2_O_2_) (Lancaster, Morecambe, UK); disodium hydrogen phosphate and sodium dihydrogen phosphate (BDH, Poole Dorset, UK); bovine serum albumin (BSA), hematoxylin monohydrate and eosin yellowish (Merck, Darmstadt, Germany); horseradish peroxidase (HRPO, Sisco Research Laboratory, Mumbai, India), dimethylformamide (DMF), tetramethylbenzidine (TMB), von Willebrand Factor (rabbit anti-human, Chemicon, Temecula, CA, USA); rabbit polyclonal inducible NOS (iNOS) and endothelial NOS (eNOS) antibodies (Santacruz Biotechnology, Delaware, USA); peroxidase conjugated anti-rabbit IgG antibody, enhanced chemiluminescence detection kit (Roche, Mannheim, Germany), PGE_2_ EIA kit, nitrate/ nitrite fluorometric assay kit, VEGF and HGF ELISA kits (Cayman Chem., Ann Arbor, MI, USA). All other chemicals were of analytical grade.

### 2.2. Preparation of GAE

Fruits of *P. emblica* L. were collected from the local market and identified by the Botanical Survey of India (Ref. no. BSI/CNH/AD/Tech./2009). The dried fruits were chopped into fine pieces, soaked in 95% ethanol for seven days and the extract was filtered through a nylon mesh. The entire process was repeated three times. The combined ethanol extracts were evaporated *in vacuo* and finally dried in a lyophilizer to obtain an amorphous brown semisolid in 10% w/w yield. The semisolid extract was stored in a vacuum dessicator.

The above extract (20.0 g) was subjected to column chromatography over silica gel (350 g), eluted with 5-100% ethyl acetate (EtOAc)/hexane and twenty-seven fractions (each of 1.0 L) were collected. All fractions were concentrated *in vacuo* and each of the fractions was tested for the DPPH (Di phenyl picryl hydrazyl) scavenging activity. The best four fractions, designated as F1-F4 obtained in 0.043, 6.08, 19.31 and 1.6% yields respectively, were used for their anti-ulcerogenic activity. Preparative thin layer chromatography (silica gel, 15 : 1.5 : 1 ethyl acetate: methanol: water) of F3 furnished pure gallic acid (65% w/w in F3). Hence it was designated as GAE and used for all the experiments. The high performance liquid chromatography (HPLC) analysis of GAE was carried out using a Zorbax 5 *μ*m C-18 column (150 × 4.6 mm) using methanol water (2 : 3 v/v; flow rate: 1.0 mL/min) as the eluent under ambient conditions. The peaks were detected at 237 nm. The major constituent was identified as gallic acid by comparing the HPLC profile of an authentic sample under the same conditions.

### 2.3. Animal

Male Swiss albino mice (6–8 weeks, 25 ± 2 g), bred in-house with free access to food and water were used for all the experiments. The mice were kept in 12-h light/dark cycles and housed at 25° ± 1°C. The animals were handled following the International Animal Ethics Committee Guidelines, ensuring minimum animal suffering. The experiments were conducted in accordance with the guidelines of the animal ethics committee of the Postgraduate Institute of Basic Medical Sciences, I.P.G.M.E&R, Kolkata (Animal Ethical Committee, Sanction No IAEC/SB-3/2008/UCM-64 Dated-15/05/08-2011).

### 2.4. Preparation of Test Samples

The test samples (GAE, gallic acid, and omeprazole) were prepared as aqueous suspensions in 2% gum acacia as the vehicle and administered to the mice orally. In some experiments, the mice were additionally treated intraperitoneally with L-NAME at the dose of 10 mg/kg, once daily for three days before drug treatment and/or L-NIL at the dose of 3 mg/kg, twice daily (first dose was administered 1 h before drug treatment and second dose was administered 15 min before drug treatment) [19].

### 2.5. Experimental Protocol for Ulceration and Assessment of Healing

The mice were divided into several groups (each containing eight mice) and each experiment was repeated four times. Except for the normal control, ulceration in the other mice was induced by indomethacin (18 mg/kg, p. o., single dose), dissolved in distilled water and suspended in 2% gum acacia as the vehicle. The animals were deprived of food but had free access to tap water 24 h, before ulcer induction. The sham treated and ulcerated untreated groups received the vehicle (0.2 ml) only throughout the course of the experiments. For the standardization of dose, GAE (3, 4, 5, 6, 7 mg/kg) was orally administered to the mice once daily up to seven days, starting the first dose 6 h after the indomethacin-administration. For comparison, treatment was also carried out with gallic acid and the positive control, omeprazole (each 3 mg/kg, p. o.). The doses of indomethacin and omeprazole were standardized in our earlier study [20, 21]. The dose of gallic acid (3 mg/kg) was decided based on its concentration in GAE. The mice were euthanized at 1st, 3rd and 7th days, four hours after the last dose of the test sample on the respective days. The stomachs from the normal and treated groups were removed rapidly, opened along the greater curvature and thoroughly rinsed with normal saline. The extent of healing was assessed from the microscopic damage scores and myeloperoxidase (MPO) activity. Additional experiments were also carried out by treating the mice with GAE or gallic acid only without indomethacin. 

### 2.6. Histology and Assay of Damage Score

The fundic portion of stomach was sectioned for histological studies as well as damage score analysis. The tissue samples were fixed in 10% formalin and embedded in paraffin. The sections (5 *μ*m) were cut using microtome, stained with hematoxylin and eosin and assessed under an Olympus microscope (BX41, Hamburg, Germany). From the histological slides, the damage scores were assessed [22] by grading the gastric injury on a 0−4 scale, based on the severity of hyperemia and hemorrhagic erosions: 0—almost normal mucosa, 0.5—hyperemia, 1—one or two lesions, 2—severe lesions, 3—very severe lesions and 4—mucosa full of lesions (lesions—hemorrhagic erosions, hyperemia—vascular congestions). The sum of the total scores divided by the mean damage score is expressed as the damage score. The experiments were performed by two investigators blinded to the groups and the treatment of animals.

### 2.7. MPO Assay

Following a reported method [23] with slight modifications, the MPO activity was determined immediately after sacrificing the animals. The whole process was carried out at 4°C. The gastric glandular portions of the stomach (100–150 mg) tissues were homogenized for 30 s in a 50 mM phosphate buffer (pH 6.0) containing 0.5% HTAB and 10 mM EDTA, followed by freezing and thawing three times. The homogenate was centrifuged at 12000 × g for 20 min at 4°C. The supernatant was collected, and the protein content was determined [24] prior to MPO assay. Then the supernatant (50 *μ*L) was added to 80 mM phosphate buffer, pH 5.4 (250 *μ*L), 0.03 M tetra methyl benzidine (TMB) (150 *μ*L) and 0.3 M hydrogen peroxide (H_2_O_2_) (50 *μ*L). After incubating the mixture at 25°C for 25 min, the reaction was terminated by adding 0.5 M sulphuric acid (H_2_SO_4_) (2.5 ml). The MPO activity was calculated from the absorbance of the mixture at 450 nm, using horseradish peroxidase (HRPO), as the standard. The MPO activity is expressed as *μ*M of H_2_O_2_ consumed per min per mg protein at 25°C and pH 5.4. 

### 2.8. Western Blot Analysis

 The glandular part of the gastric mucosa after being washed with PBS containing protease inhibitors was minced and homogenized in a lysis buffer (10 mM Tris-HCl pH 8.0, 150 mM NaCl, 1% Triton X-100) containing aprotinin (2 *μ*g/ml), leupeptin (2 *μ*g/ml) PMSF (0.4 *μ*M), and type II phosphatase inhibitor. Following centrifugation at 15,000 × g for 30 min at 4°C, the supernatant was collected, aliquoted and kept at −70°C prior to use for the western blots. The protein concentration was measured [24]. The proteins (40 *μ*g) were resolved by 10% SDS-polyacrylamide gel electrophoresis and transferred to nitrocellulose membrane. The membrane was blocked for 2 h in TBST buffer (20 mM Tris-HCl, pH 7.4, 150 mM NaCl, 0.02% Tween 20) containing 99% fat-free milk powder (5%) and incubated overnight at 4°C with rabbit polyclonal iNOS or eNOS antibody (1 : 2000 dilution). The membrane was washed over a period of 2 h with TBST and incubated with peroxidase conjugated anti-rabbit IgG (1 : 2500 dilution). The bands were detected using an enhanced chemiluminescence detection kit and quantified with respect to that of bands of a suitable loading control, using the Kodak Gelquant software. 

### 2.9. Protein Content Assay

The protein content was determined by the Bradford method using BSA as the standard [24].

### 2.10. PGE_2_ Assay

The PGE_2_ level in tissue homogenate was estimated using commercially available ELISA kit (Cayman Chemical, Ann Arbor, MI, USA), following manufacturer's protocol. The stomach was excised, weighed (100 mg) and suspended in 10 mM sodium phosphate buffer, pH 7.4 (1 mL). The tissues were finely minced and incubated at 37°C for 20 min. After centrifugation (9000 × g), the PGE_2_ level in the supernatant was measured by ELISA, following manufacturer's instructions. The samples along with the standards were seeded to each well at an appropriate dilution and PGE2 express AChE tracer and PGE2 express monoclonal antibody (both AChE tracer and monoclonal antibody supplied in kit) were added. The plate was covered and incubated for 60 min at room temperature on an orbital shaker. The wells were washed (5 times), Ellman's reagent to each well was added, and the mixture was incubated further for 60–90 min at dark. Next, absorbance was read at wavelength 405 nm.

### 2.11. Tissue NO Assay

In aqueous medium, cellular NO is rapidly converted to nitrite and nitrate. However, their ratio varies substantially depending on the environment. Hence, for this assay, we used a nitrate/nitrite fluorometric assay kit (Cayman Chem., Ann Arbor, MI). In brief, tissue samples were homogenized in PBS (pH 7.4) and centrifuged at 10,000 × g for 20 min at 4°C. The supernatant was filtered through a 0.45 micron filter (Millipore, Leiden, the Netherlands) and the filtrate was filtered through a 10 kDa molecular weight cutoff ultrafiltration unit (Millipore, Leiden, the Netherlands). The filtrate was assayed spectrofluorimetrically using the fluorescent dye 2, 3-diaminonaphthalene (DAN, excitation wavelength = 365 nm and emission wavelength = 430 nm), following manufacturer's protocol [25].

### 2.12. Quantification of von Willebrand Factor VIII

The number of microvessels were assessed from von Willebrand Factor VIII, following a reported procedure [19] with slight modifications. In brief, after deparaffinization and rehydration, the endogenous peroxidase activity in the tissue was blocked with 0.3% hydrogen peroxide in methanol. The tissue sections were incubated with the polyclonal rabbit anti-human von Willebrand Factor VIII antibody for 2 h at room temperature and the bound primary antibody was detected using the cell and tissue staining kit. Any positive-staining endothelial cell or endothelial cell cluster that was clearly separated from adjacent microvessels was considered as an angiogenic microvessel. The vascular areas immediately adjacent to the normal tissue of the stomach served as the internal control. The microvessels (under 10X magnification) in five randomly selected microscopic fields of mucosal erosions were counted in a blinded manner and the data were averaged.

### 2.13. Estimation of Tissue Growth Factors

The tissue VEGF and HGF levels were estimated using commercially available ELISA kits (Cayman Chemical, Ann Arbor, MI).

### 2.14. Statistical Analysis

Data are expressed as mean ± S.E. unless mentioned otherwise. Values of the band intensity of the immunoblots (arbitrary unit, mean ± S.E.M.) are the density scanning results of three independent experiments, considering that of normal mice as 1. Comparisons were made between different treatments using one-way analysis of variance (ANOVA) followed by an error protecting multiple comparison procedure, namely, Tukey-Kramer post hoc test by Graph Pad InStat (GraphPad Software Inc., San Diego, USA) software for the analysis of significance of all the data.

## 3. Results

### 3.1. Assessment of Ulcer Healing

**Figure 1:**
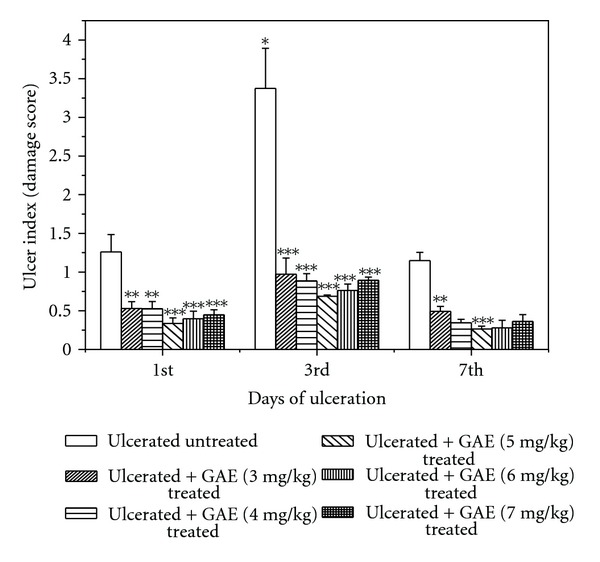
Healing capacities of GAE under various treatment regimes against indomethacin-induced acute gastric mucosal injury in mice. Ulceration in the mice was induced by indomethacin (18 mg /kg, single dose, p.o.). Treatment was carried out with GAE (3, 4, 5, 6, 7 mg/kg, single dose daily up to 7 days, p. o.) after indomethacin administration. The section of mice stomachs were dissected on the 1st, 3rd, and 7th days of ulceration, 4 h after the last dose of the test sample, and the damage scores of different mice groups were measured. The values are mean ± S.E. of four independent experiments, each with 8 mice/group. *P < 0.001, compared to 1st day ulcerated mice; **P < 0.01, ***P < 0.001, compared to untreated mice of the same day.

**Figure 2:**
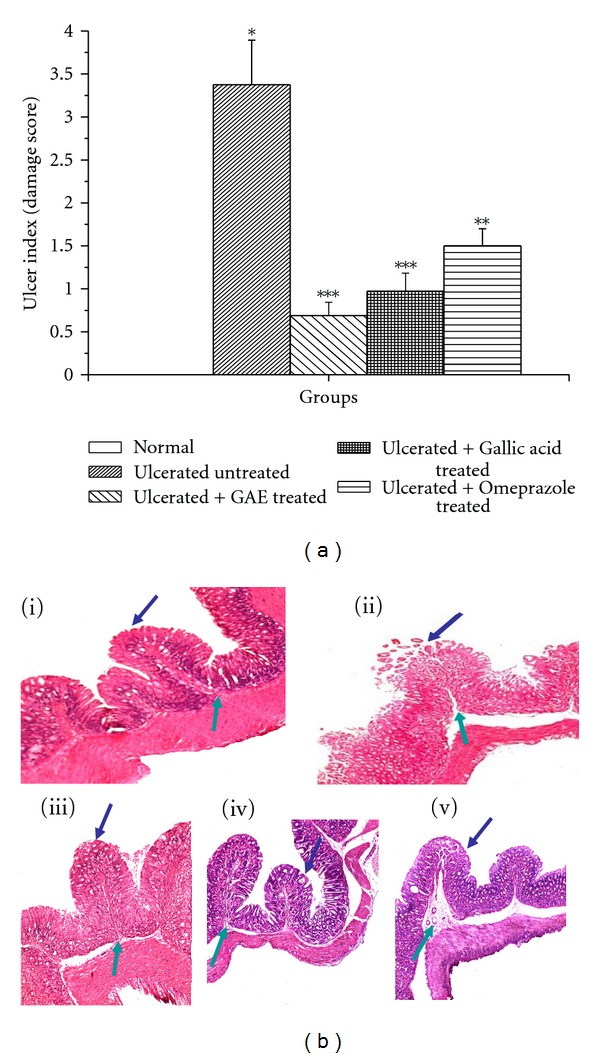
Healing capacities of GAE, gallic acid and omeprazole under the optimized treatment regime. (a) ulcer indices; (b) histology. Ulceration in the mice was induced by indomethacin (18 mg/kg, single dose, p.o.). Treatment was carried out with GAE (5 mg/kg daily, p.o.), gallic acid (3 mg/kg daily, p.o.), and omeprazole (3 mg/kg daily, p.o.) for 3 days, starting the first dose 6 h postulcer induction. The sections of mice stomachs were processed for capturing the images. Representative histology of gastric tissue sections are shown at 10x magnification. (i) normal, (ii) Ulcerated untreated, (iii) Ulcerated + GAE treated, (iv) Ulcerated + Gallic acid treated, (v) Ulcerated + Omeprazole treated, mucosal, and submucosal layers are shown by blue and green arrows, respectively. The ulcer indiceswere calculated from the damage scores.The values are mean ± S.E. of four independent experiments, each with 8 mice/group. *P < 0.001, compared to normal mice; **P < 0.01,***P < 0.001, compared to untreated mice.

Indomethacin (18 mg/kg, p.o., single dose) administration produced acute time-dependent mucosal lesions in the mice stomach, as evident from histology. Quantification of the damage scores on the respective days revealed maximum ulcerative damage on the 3rd day of indomethacin administration. However, the ulcerative damage reduced on the 7th day. Amongst the chosen doses of GAE, best ulcer healing was observed at a dose of 5 mg/kg, irrespective of the day of ulceration (Figure 1). The healing capacities of GAE at its optimized dose (5 mg/kg daily × 3 days, p.o.) and pure gallic acid (3 mg/kg daily × 3 days, p.o.) are shown in stomach histology (Figure 2(b)) and damage scores (Figure 2(a)). Compared to the untreated group, the damage scores in the GAE, gallic acid and omeprazole-treated groups were reduced by 79.6%, 71.2% and 55.9%, respectively. Mice receiving only vehicle did not produce any gastric lesion. On their own, GAE and gallic acid were found to be nonulcerogenic.

### 3.2. Regulation of Mucosal MPO Activity

**Figure 3:**
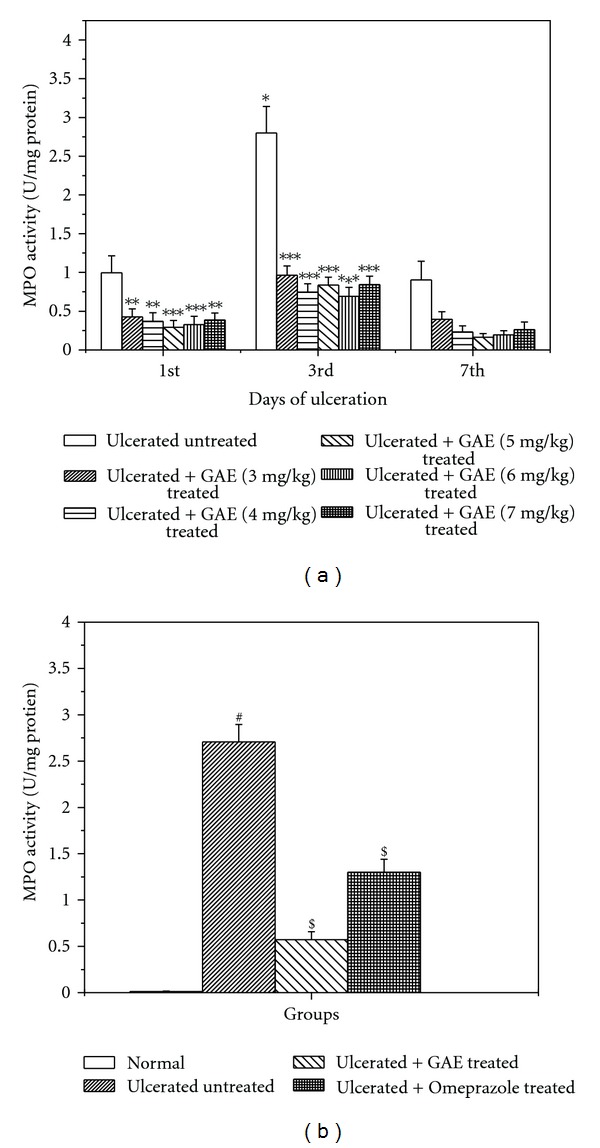
Reduction of the mucosal MPO activity in ulcerated mice by GAE. (a) under various treatment regimes; (b) optimized treatment regime. Ulceration in the mice was induced by indomethacin (18 mg/kg, single dose, p.o.). Treatment was carried out with different doses of GAE upto 7 days after indomethacin (18 mg/kg, single dose, p.o.) administration. The section of mice stomachs were dissected on the 1st, 3rd, and 7th days of ulceration, 4 h after the last dose of the test sample, and the MPO activities of different groups of mice were measured. Omeprazole (3 mg/kg × 3 days, p. o.) was used as the positive control. The values are mean ± S.E. of four independent experiments, each with 8 mice/group. *P < 0.001, compared to 1st day ulcerated mice; **P < 0.01, ***P < 0.001, compared to untreated mice of the same day. ^#^ *P* < 0.001, compared to 3rd day normal mice; ^$^ *P* < 0.001, compared to 3rd day untreated mice.

The mucosal MPO activity of the indomethacin-administered mice increased immediately, reaching the peak value on the 3rd day, and thereafter declining on the 7th day of ulceration (Figure 3(a)). The results were consistent with the damage score data. Treatment with GAE (5 mg/kg daily, p.o.) and omeprazole (3 mg/kg daily, p.o.) for 3 days reduced the MPO activity by 78.8% and 51.8%, respectively, compared to that of the untreated group (Figure 3(b)). 

### 3.3. Regulation of PGE_2_ Synthesis

**Figure 4:**
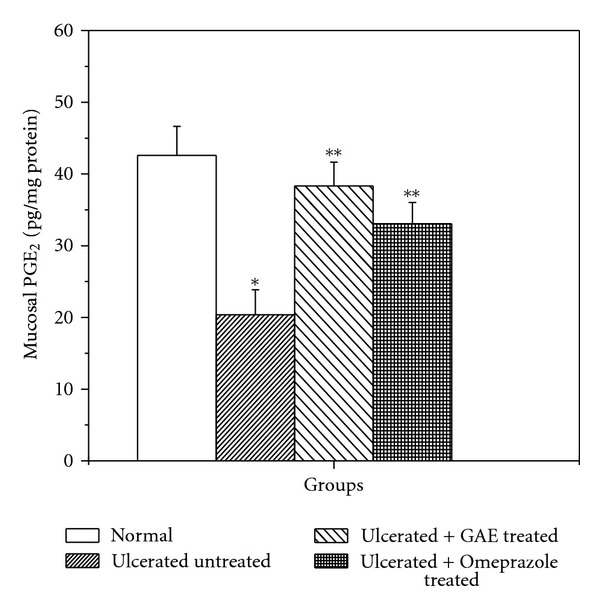
Effect of GAE on mucosal PGE_2_ synthesis in indomethacin-induced ulcerated mice. Ulceration in the mice was induced by indomethacin (18 mg/kg, single dose, p.o.). Treatment was carried out for 3 days with GAE (5 mg/kg, daily, p. o.) or omeprazole (3 mg/kg, p. o.) after ulcer induction. The mucosal PGE_2_ levels of the ulcerated untreated and treated mice were measured. The values are mean ± S.E. of four independent experiments, each with 8 mice/group. *P < 0.001, compared to normal mice; **P < 0.001, compared to untreated mice.

Compared to the normal control, the mucosal PGE_2_ level was markedly suppressed (2.1 fold) in the untreated mice. Treatment with GAE and omeprazole for three days upregulated the mucosal PGE_2_ level by 88.2% and 65% respectively, compared to the untreated group (Figure 4).

### 3.4. Modulation of NOS Expression

**Figure 5:**
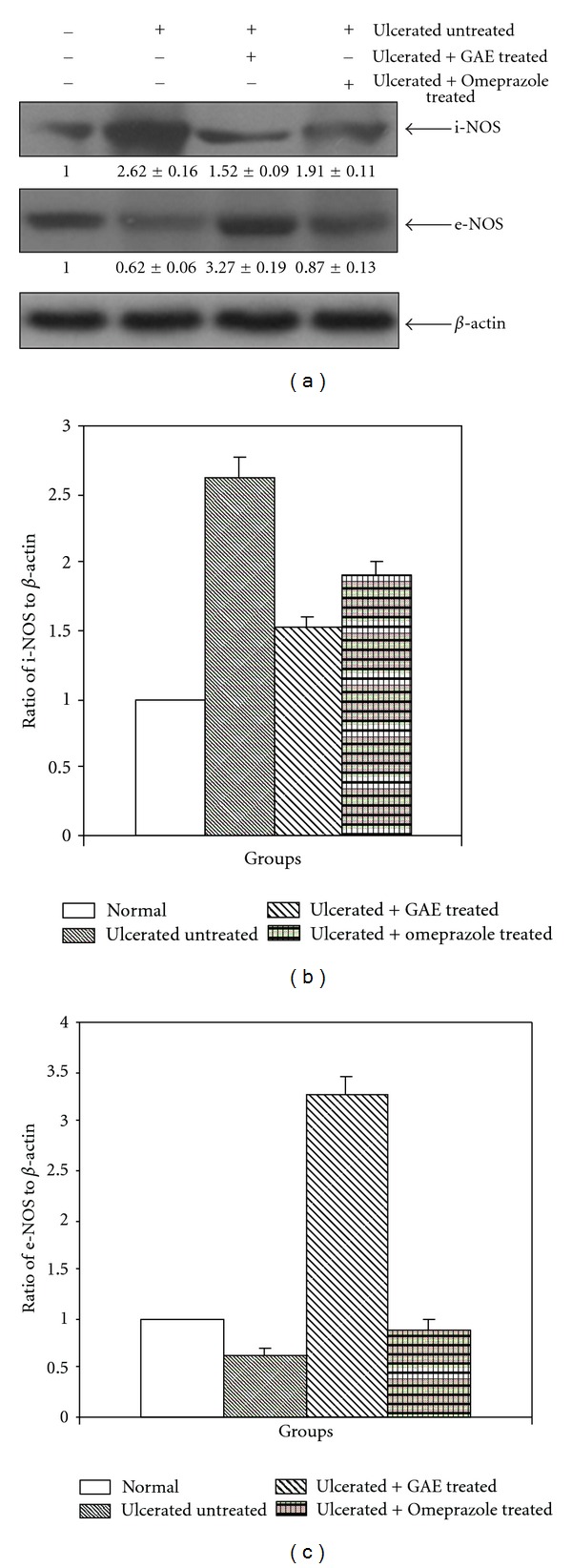
The e-NOS and i-NOS expressions in normal, ulcerated and GAE (5 mg/kg, single dose daily × 3 days, p. o.) or omeprazole (3 mg/kg, p. o.) treated gastric tissues of mice, and their quantifications. Western blots of the expressions of the enzymes (A). Ratios of the intensities of i-NOS (B) and e-NOS (C) bands to that of the respective *β*-actin bands as quantified from the western blot images, using Kodak Gelquant software. The values (arbitrary unit, mean ± S.E.M.) are the density scanning results of three independent experiments, considering that of normal mice as 1.

The Western blots of e-NOS and i-NOS expressions in the gastric mucosa of the control, ulcerated and drug (GAE or omeprazole)-treated mice are shown in Figure 5. The e-NOS expression was detected in both normal and ulcerated gastric tissues. In contrast, the i-NOS expression was very high in the ulcerated tissues, but much less in normal gastric tissues. Our western blot data revealed that three-day treatment with GAE significantly induced e-NOS expression, while reducing i-NOS expression, compared to that in the untreated group. Although omeprazole also made similar changes in the expressions of the enzymes, however, the effect was much less.

### 3.5. Modulation of Tissue NO Level

**Figure 6:**
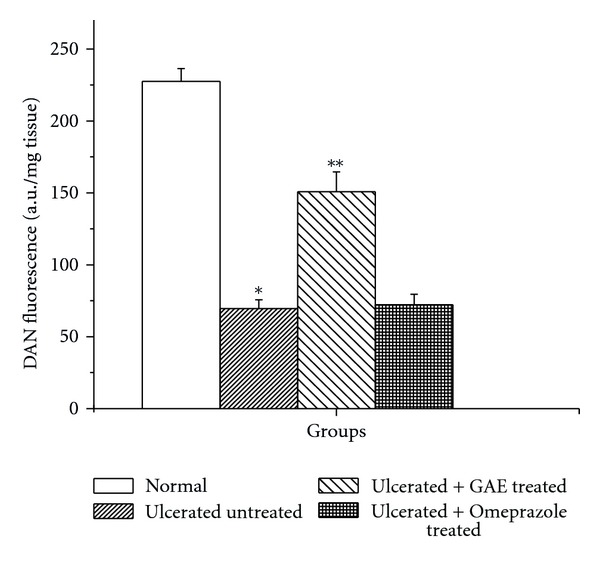
Effect of GAE on mucosal NO level in indomethacin-induced ulcerated mice. Ulceration in the mice was induced by indomethacin (18 mg/kg, single dose, p. o.). Treatment was carried out for 3 days with GAE (5 mg/kg, single dose daily × 3 days, p. o.) or omeprazole (3 mg/kg, p. o.) after ulcer induction. The mucosal NO levels of the ulcerated untreated and treated mice were measured. The values are mean ± S.E. of four independent experiments, each with 8 mice/group. *P < 0.001, compared to normal mice; **P < 0.001, compared to untreated mice.

Compared to the normal control group, the tissue NO level in the ulcerated untreated mice was suppressed by 69.4% (Figure 6). Compared to the untreated mice, the tissue NO level was markedly increased (2.2 fold) in the GAE-treated group, while omeprazole did not significantly alter this.

### 3.6. Quantification of von Willebrand Factor (vWF) VIII

**Figure 7:**
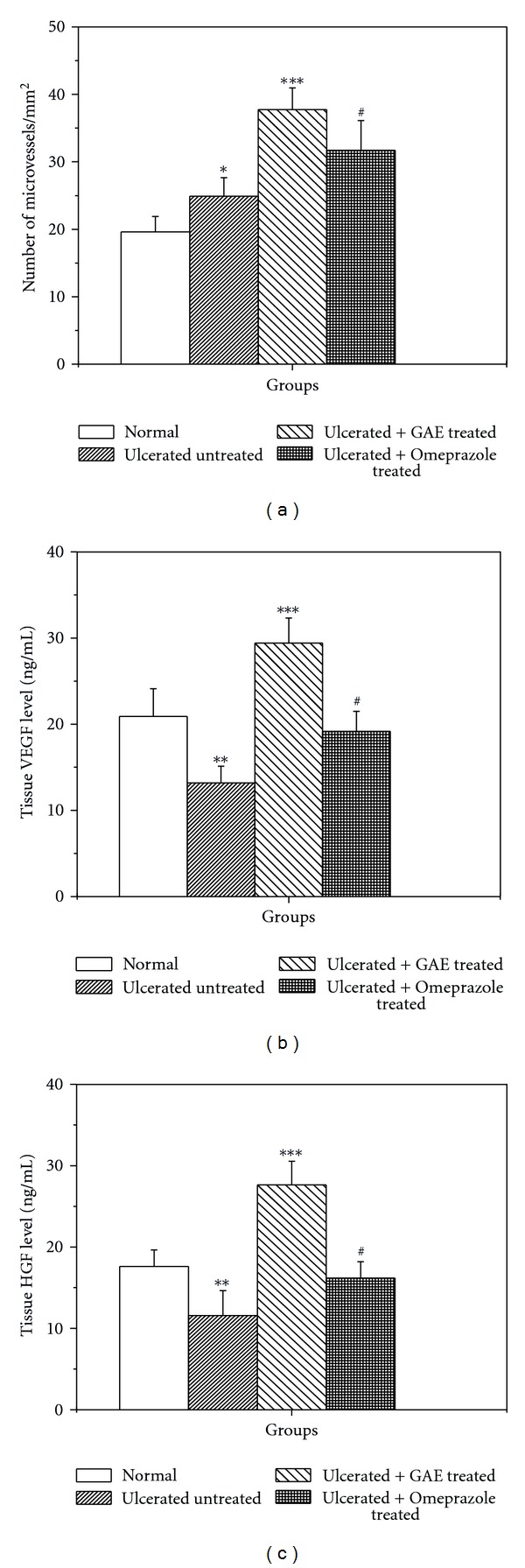
Effect of GAE on different angiogenic parameters in indomethacin-induced ulcerated mice. (a) von Willebrand Factor VIII; (b) mucosal VEGF level; (c) mucosal HGF level. Ulceration in the mice was induced by indomethacin (18 mg /kg, single dose, p. o.). Treatment was carried out for 3 days with GAE (5 mg/kg, single dose daily × 3 days, p. o.) or omeprazole (3 mg/kg, p. o.) after ulcer induction. The mucosal von Willebrand Factor VIII (expressed as number of microvessels/mm^2^) and growth factors (expressed as ng/ml tissue extract) were measured by immunohistochemistry and colorimetry, respectively. *P < 0.05, **P < 0.01, compared to normal mice; ^#^ *P* < 0.01, ***P < 0.001, compared to untreated mice.

The microscopic results using immunostaining of the von Willebrand Factor VIII revealed the presence of 19.6 ± 1.15 microvessels/mm^2^ in submucosa of control mice. This increased to 24.9 ± 1.42 in the ulcerated untreated mice (Figure 7(a)). Treatment with GAE and omeprazole-enhanced the microvessel number by 51.6% and 27.3% respectively compared to that in the untreated mice. 

### 3.7. Regulation of Growth Factors

Indomethacin administration downregulated the VEGF and HGF levels by 36.8% and 34.2%, respectively, compared to sham-treated mice. The VEGF and HGF levels in the GAE-treated group were increased 2.2-fold and 2.4-fold, respectively, compared to the untreated mice (Figures 7(b) and 7(c)). Omeprazole increased the VEGF and HGF levels by 45.1% and 39.6%, respectively, compared to the untreated mice.

### 3.8. Effect of NOS Inhibitors on the Healing Property of GAE

**Figure 8:**
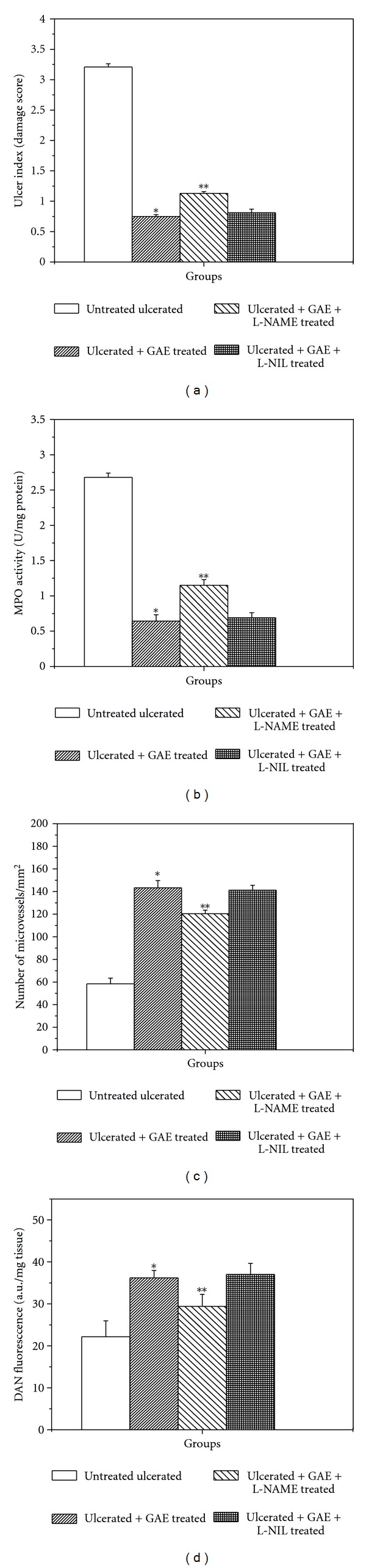
Effect of NOS inhibitors on the healing activity of GAE in indomethacin-induced ulcerated mice. (a) damage score, (b) MPO activity, (c) von Willebrand Factor VIII, (d) NO level. Ulceration in the mice was induced by indomethacin (18 mg /kg, single dose, p.o.). After ulcer induction, treatment was carried out with GAE (5 mg/kg, single dose daily, p. o.) alone or in conjunction with L-NAME (15 mg/kg, once daily) or L-NIL (3 mg/kg, twice daily) for 3 days. The parameters of the ulcerated untreated and treated mice were measured. *P < 0.001 compared to normal mice; **P < 0.01, compared to GAE-treatment.

Treatment with L-NAME in the GAE-treated group significantly increased the damage score (50.7%) (Figure 8(a)) and MPO activity (78.9%) (Figure 8(b)), but reduced the mucosal NO level by 1.1-fold (Figure 8(c)) and microvessels numbers by 1.2-fold (Figure 8(d)) compared to the sole GAE-treated group. But none of these parameters changed significantly in the GAE + L-NIL group, compared to the GAE-treated group.

## 4. Discussion

Several factors such as oxidative stress, neutrophils activation, as well as modulation of various enzymes, cytokines and soluble mediators play crucial roles in the indomethacin-mediated gastric ulceration and delayed ulcer healing [26]. Controlling these factors provides an opportunity to develop improved antiulcer medications. The gastrotoxicity of indomethacin is generally explained in terms of COX inhibition, reduced PG synthesis and the impaired PG-mediated angiogenesis. However, the process also involves alternate COX-independent mechanisms, wherein other contributors such as the nitrogen-metabolizing enzymes [13, 14] and neutrophil infiltration [27] determine the healing process. 

The impressive healing capacity of the ethanolic extract of amla against the indomethacin-induced gastric ulcer [15] encouraged us to investigate the probable modulatory effect of the extract on the COX-dependent [28] and independent pathways [29] of wound healing. For this purpose, we used the gallic acid-enriched fraction (GAE) of the amla extract. In the present study, indomethacin administration led to mucosal damage and augmented the MPO activity in the ulcerated area of the gastric wall. Because MPO activity is increased by the activated neutrophils, the above results suggested the involvement of neutrophils infiltration in gastric ulceration. The MPO activity is known to increase under the ulcerated conditions, and reduced during the healing process [30]. It is often used as a risk marker and diagnostic tool for assessing severity of gastric ulcer [31]. Treatment with GAE could sufficiently restore the normal gastric mucosal integrity, while reducing the MPO activity. Earlier, the crude ethanolic extract of amla (60 mg/kg) showed similar healing activity as that of GAE (5 mg/kg). However, the GAE content of the crude extract (60 mg/kg) would be ~11 mg. Further, the extract was marginally more potent than pure gallic acid at the concentrations present in the effective dose of GAE. Taken together, these results established that gallic acid is the active constituent of GAE, but some other constituents of GAE may play synergistic roles in healing activity of GAE. These results also suggested a close relationship between the state of the gastric inflammation and MPO activity. Earlier pretreatment with an antibody against neutrophils was found to prevent the indomethacin-induced gastric ulceration [32]. Based on these, it is tempting to propose that indomethacin first stimulates the neutrophils to release substances which are related to inflammation. However, further studies are needed to clarify the sequence of events. 

Besides indicating ulcer initiation and progression, neutrophils infiltration is also reported to delay gastric ulcer healing [33] and its reduction accelerates ulcer healing [34]. Oxygen-free radicals derived from the activated neutrophils delay gastric ulcer healing in rats [35]. Furthermore, neutrophils infiltration induces microcirculatory abnormalities [36] and its suppression promotes healing [37]. Hence, we used it as an oxidative marker in the present study. 

The NSAIDs exert both therapeutic and toxic effects, mainly through reduction of the levels of circulating PGE_2_ at the gastric mucosa. Besides stimulating mucus and bicarbonate secretion and mucosal blood flow, PGs also contribute to ulcer healing by inducing angiogenesis [17]. The reduced PGE_2_ causes gastric ulceration and also exacerbates preexisting gastric ulcers in rodents and human [25]. Our data showed that indomethacin treatment depleted the tissue PGE_2_ level that was increased significantly in the drug-treated groups (Figure 4). The effect of GAE was better than that of omeprazole. Enhanced PG synthesis is known to inhibit neutrophils-mediated free radical generation [38]. Therefore, stimulation of PGE_2_ level by GAE might contribute to its antioxidative property, observed in the previous study.

The physiologically important NO, produced during arginine catabolism by the NOSs plays dual roles in gastric mucosal defense and injury. The low concentration of NO, produced by e-NOS, one of the constitutive NOS isoforms helps in wound healing by increasing blood flow [39] and angiogenesis [19, 40] in the damaged gastric mucosa. However, its enhanced generation by i-NOS may contribute to the pathogenesis of various gastroduodenal disorders including peptic ulcer [30]. An increase in i-NOS activity and a decrease in e-NOS activity in the gastric mucosa are closely related to the development of gastric mucosal lesions. Currently we confirmed that the indomethacin-induced gastric ulceration increased the mucosal i-NOS expression, but reduced the e-NOS expression in mice. Piotrowski et al. [41] showed a 12-fold increase in gastric epithelial expression of iNOS activity in the indomethacin-administered animals, compared to controls and the increase correlated positively with the epithelium damage. Our results showed only 1.3-fold increased i-NOS expression after ulceration. This may possibly be due to the fact that we assayed it on the 3rd day of ulceration. Despite the increased i-NOS expression, the tissue NO level was significantly reduced in the indomethacin group. The apparent discrepancy may be due the fact that i-NOS expression itself may not match with its activity. Also, the generated NO may be scavenged through NADPH oxidase or MPO catalyzed reactions [42]. The reduction of the beneficial vasodilatory NO would delay the ulcer healing. Treatment with GAE raised the e-NOS/i-NOS ratio to a level favourable for efficient ulcer healing. The associated increase in the tissue NO level must be derived through the e-NOS-catalyzed reaction, because GAE increased e-NOS, but not i-NOS expressions. Earlier, using e-NOS deficient mice, the importance of e-NOS and e-NOS-derived NO in regulating microvascular structure during acute inflammation has been demonstrated [43]. 

Gastric ulcer healing entails several distinct repair mechanisms. The epithelial cell proliferation and migration from the ulcer edge across the ulcer bed is accompanied by maturation of granulation tissue beneath the ulcer base. Within this tissue vascular endothelial cells form new capillaries to restore the microvasculature, while fibroblasts restore the lamina propria. The degree of neovascularisation (angiogenesis), assessed by specific endothelial markers including von Willebrand Factor VIII, CD31, and CD34 in experimental ulcer models correlates well with the extent and speed of ulcer healing. Among these markers, von Willebrand Factor VIII acts as a cofactor for platelet binding to expose extracellular matrix in injured vessel walls. A large number of factors including several growth factors regulate angiogenic wound healing at its various stages [18, 44, 45]. Amongst these, VEGF triggers endothelial proliferation and migration and accelerate ulcer healing by promoting angiogenesis [14, 46]. Likewise HGF, expressed at the ulcer margin to act as trophic factors for the gastric mucosa helps angiogenesis by multiple mechanisms including COX activation [47]. Hence, we focused on these growth factors for the present studies.

Our result on the increased number of mucosal von Willebrand Factor VIII in the ulcerated mice over that of normal control mice is consistent with the requirement of more microvessels for ulcer healing. The increased number of microvessels would assist better blood flow and transport of oxygen and nutrients to the site of inflammation for quicker healing. Treatment with GAE increased the von Willebrand Factor VIII further. This explains the accelerated ulcer healing by GAE, compared to natural healing. The results are consistent with our previous finding with a resveratrol-analogue that also increased the e-NOS/i-NOS ratio to provide better angiogenesis [25]. Indomethacin inhibits ADP-induced platelet aggregation and release of the *α*-granule, which stores VEGF. Consequently, indomethacin treatment would reduce VEGF release. We also found that indomethacin administration suppressed the levels of VEGF and HGF. Both these parameters were increased significantly beyond the respective normal values by GAE treatment (Figures 7(b) and 7(c)). 

Enhanced synthesis of mucosal PGE_2_ and e-NOS-derived NO by GAE might be instrumental in their ulcer-healing action. On the other hand, omeprazole did not show any significant effect on NO synthesis (data not shown). 

To substantiate our hypothesis that modulation of e-NOS may primarily account for the excellent ulcer healing capacity of GAE, we studied the effects of L-NIL, a specific i-NOS inhibitor and L-NAME, a nonspecific NOS inhibitor on the healing capacities of GAE. For this, we assessed four different parameters, namely, (i) ulcer index, (ii) MPO activity, (iii) von Willebrand Factor VIII, and (iv) tissue NO level of the GAE-treated mice in the absence and presence of the above inhibitors. Since i-NOS expression was effectively inhibited by GAE alone (Figure 5), addition of L-NIL did not alter any of these parameters significantly. However, addition of L-NAME would suppress both e-NOS and i-NOS expressions, negating the augmented e-NOS expression, caused by GAE. Consistent with this, treatment with GAE in conjunction with L-NAME led to increased ulcer index and MPO activity with associated decrease in von Willebrand Factor VIII and tissue NO level, compared to that with the only GAE-treated mice (Figure 8). Taken together our results established that the e-NOS-derived NO contributed maximum to the ulcer healing property of GAE, although a role for neuronal NOS-derived NO cannot be excluded.

## 5. Conclusion

**Figure 9:**
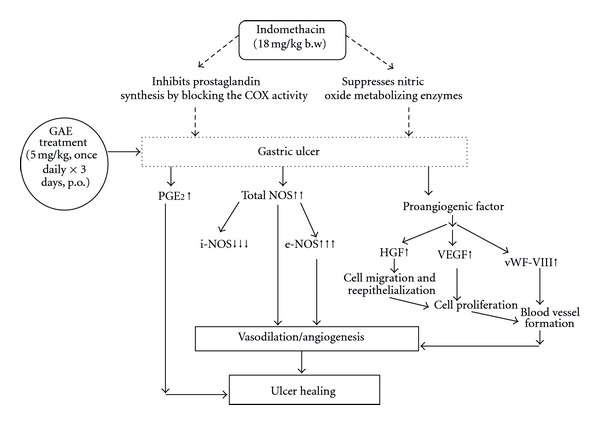
Schematic representation of the plausible mechanism of the ulcer healing by GAE.

Overall, gallic acid was the active principle of the gallic acid enriched ethanolic amla extract (GAE) that promoted healing of indomethacin-induced gastric ulcers in mice. The beneficial effect of GAE was due to its ability to reduce neutrophils infiltration and increase mucosal PGE_2_ as well as NO levels that were downregulated by indomethacin. GAE increased the mucosal NO by augmenting the e-NOS/i-NOS ratio. All these factors, especially the modulation of the NOS-pathway helped in upregulating mucosal VEGF and HGF levels to promote angiogenesis and accelerate ulcer healing (Figure 9). Our present and previous results with amla extract, coupled with its nontoxic nature, suggest GAE as a promising antiulcerogenic formulation and opened the way for further evaluation. 

## Supplementary Material

Supplementary Figure 1: Effect of GAE on total antioxidant status (A) and lipid peroxidation (B) in indomethacininduced ulcerated mice. Ulceration in the mice was induced by indomethacin (18 mg/kg, single dose). Treatment was carried out for 3 days with GAE (5 mg/kg, daily) or omeprazole (3 mg/kg) after ulcer induction.
Supplementary Figure 2: Effect of L-NAME in indomethacin -induced ulcerated mice. A: damage score, B: MPO activity, C: von Willebrand Factor VIII, D: NO level. Ulceration in the mice was induced by indomethacin (18 mg/kg, single dose). After ulcer induction, treatment was carried out with L-NAME (15 mg/kg, once daily) for 3 days.

## Abbreviations

GAE: Gallic acid enriched fraction
NSAID: Nonsteroidal anti-inflammatory drug
PG: Prostaglandin
VEGF: Vascular endothelial growth factor
HGF: Hepatocyte growth factor
L-NIL: L-*N*6-(1-iminoethyl) lysine hydrochloride
L-NAME: *N*-nitro-L-arginine methyl ester.
