# Supplementary material for: Gallic Acid Enriched Fraction of Phyllanthus emblica Potentiates Indomethacin-Induced Gastric Ulcer Healing via e-NOS-Dependent Pathway
Source: Evid Based Complement Alternat Med. 2012 Aug 26;2012:487380. doi: 10.1155/2012/487380 (PMC3433150; doi:10.1155/2012/487380)
Supplement: Supplementary file 1 — Supplementary Figure 1: Effect of GAE on total antioxidant status (A) and lipid peroxidation (B) in indomethacininduced ulcerated mice. Ulceration in the mice was induced by indomethacin (18 mg/kg, single dose). Treatment was carried out for 3 days with GAE (5 mg/kg, daily) or omeprazole (3 mg/kg) after ulcer induction. Supplementary Figure 2: Effect of L-NAME in indomethacin -induced ulcerated mice. A: damage score, B: MPO activity, C: von Willebrand Factor VIII, D: NO level. Ulceration in the mice was induced by indomethacin (18 mg/kg, single dose). After ulcer induction, treatment was carried out with L-NAME (15 mg/kg, once daily) for 3 days. [file 487380.f1.pdf]

Supplementary Figure: 1

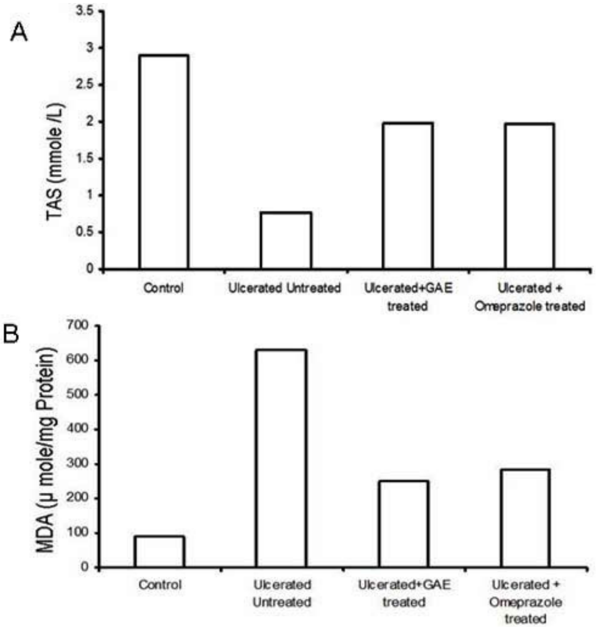

Supplementary Figure:1

Effect of GAE on total antioxidant status (A) and lipid peroxidation (B) in indomethacin-induced ulcerated mice. Ulceration in the mice was induced by indomethacin (18 mg /kg, single dose). Treatment was carried out for 3 days with GAE (5 mg/kg, daily) or omeprazole (3 mg/kg) after ulcer induction.

Supplementary Figure:2

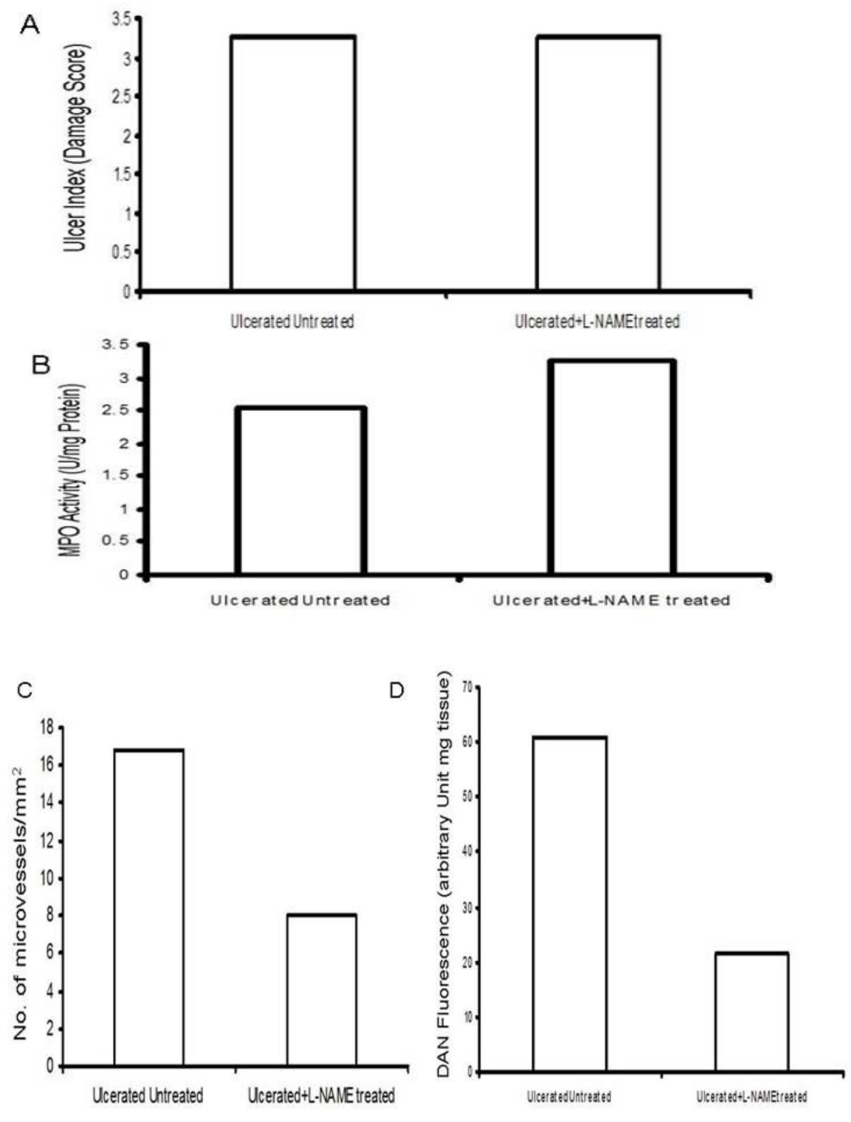

Supplementary Figure:2

Effect of L-NAME in indomethacin -induced ulcerated mice. **A**: damage score, **B**: MPO activity, **C**: von Willebrand Factor VIII, **D**: NO level. Ulceration in the mice was induced by indomethacin (18 mg /kg, single dose). After ulcer induction, treatment was carried out with L-NAME (15 mg/kg, once daily) for 3 days.
